# Aerobic Swim Training Restores Aortic Endothelial Function by Decreasing Superoxide Levels in Spontaneously Hypertensive Rats

**DOI:** 10.6061/clinics/2017(05)09

**Published:** 2017-05

**Authors:** Camila P Jordão, Tiago Fernandes, Leonardo Yuji Tanaka, Luiz R. Grassmann Bechara, Luis Gustavo Oliveira de Sousa, Edilamar M Oliveira, Paulo Rizzo Ramires

**Affiliations:** I Laboratorio de Bioquimica e Biologia Molecular do Exercicio, Escola de Educacao Fisica e Esporte, Universidade de Sao Paulo, Sao Paulo, SP, BR; II Laboratorio de Biologia Vascular, Instituto do Coracao (InCor), Hospital das Clinicas HCFMUSP, Faculdade de Medicina, Universidade de Sao Paulo, Sao Paulo, SP, BR; III Unidade de Reabilitação, Instituto do Coracao (InCor), Hospital das Clinicas HCFMUSP, Faculdade de Medicina, Universidade de Sao Paulo, Sao Paulo, SP, BR

**Keywords:** Aerobic Training, Vasodilation, Nitric Oxide, Superoxide, Hypertension

## Abstract

**OBJECTIVE:**

We aimed to determine whether aerobic training decreases superoxide levels, increases nitric oxide levels, and improves endothelium-dependent vasodilation in the aortas of spontaneously hypertensive rats.

**METHODS:**

Spontaneously hypertensive rats (SHR) and Wistar Kyoto rats (WKY) were distributed into 2 groups: sedentary (SHRsd and WKYsd, n=10 each) and swimming-trained (SHRtr, n=10 and WKYtr, n=10, respectively). The trained group participated in training sessions 5 days/week for 1 h/day with an additional work load of 4% of the animal’s body weight. After a 10-week sedentary or aerobic training period, the rats were euthanized. The thoracic aortas were removed to evaluate the vasodilator response to acetylcholine (10^-10^ to 10^-4^ M) with or without preincubation with L-N^G^-nitro-L-arginine methyl ester hydrochloride (L-NAME; 10^-4^ M) *in vitro*. The aortic tissue was also used to assess the levels of the endothelial nitric oxide synthase and nicotinamide adenine dinucleotide oxidase subunit isoforms 1 and 4 proteins, as well as the superoxide and nitrite contents. Blood pressure was measured using a computerized tail-cuff system.

**RESULTS:**

Aerobic training significantly increased the acetylcholine-induced maximum vasodilation observed in the SHRtr group compared with the SHRsd group (85.9±4.3 *vs*. 71.6±5.2%). Additionally, in the SHRtr group, superoxide levels were significantly decreased, nitric oxide bioavailability was improved, and the levels of the nicotinamide adenine dinucleotide oxidase subunit isoform 4 protein were decreased compared to the SHRsd group. Moreover, after training, the blood pressure of the SHRtr group decreased compared to the SHRsd group. Exercise training had no effect on the blood pressure of the WKYtr group.

**CONCLUSIONS:**

In SHR, aerobic swim training decreased vascular superoxide generation by nicotinamide adenine dinucleotide oxidase subunit isoform 4 and increased nitric oxide bioavailability, thereby improving endothelial function.

## INTRODUCTION

Arterial hypertension (AH) is a highly prevalent disease that affects a large proportion of the global population 1 and is a critical risk factor for the development of cardiovascular diseases (CVDs) 2. AH is associated with the impairment of endothelium-dependent vasodilation, an important characteristic of endothelial dysfunction 3,4. The endothelium has a crucial role in the control of vascular tonus because of its ability to produce both relaxing and constricting factors, such as nitric oxide (NO) and superoxide, respectively (5,6). NO is the most widely studied and is the major endothelium-derived relaxing factor 7,8. Reduced NO levels are associated with endothelial dysfunction 5. A reduction in endothelium-derived NO bioavailability has been suggested to be responsible for endothelial dysfunction in patients with hypertension for the following two possible reasons: 1) decreased NO production by endothelial nitric oxide synthase (eNOS) 9; and 2) increased levels of vascular superoxide, which increase NO degradation 5.

Superoxide has been shown to play a decisive role in the control of vascular function 10. Among other sources, superoxide is produced from the single electron reduction of oxygen by nicotinamide adenine dinucleotide phosphate (NADPH) oxidase in arterial vessels 11,12. Because NADPH oxidase exclusively produces superoxide, it has been identified as a major source of superoxide generation in the vascular system 13. However, at least 3 catalytic NADPH oxidase subunits are expressed in the rodent vasculature, i.e., Nox1, Nox2 and Nox4 (NAD(P)H oxidase subunit homologues of gp91phox), the last of which appears to have the highest expression levels of mRNA, especially in the aorta 11,14. In addition, the Nox4 and Nox1 subunits are the most abundant in vascular smooth muscle cells, and their mRNA expression is regulated by angiotensin II 11,14. In both humans with hypertension and in SHR, both local and systemic renin-angiotensin-aldosterone systems are activated, which leads to increased expression of the Nox4 and/or Nox1 proteins and, consequently, to a pro-oxidant state resulting from chronically increased superoxide production 15-17.

Under physiological conditions, superoxide is produced at low concentrations, and it works as a signaling molecule 18,19. Conversely, in subjects with hypertension, increased superoxide production leads to a decrease in NO availability and may promote endothelial dysfunction 20,21.

Thus, many forms of intervention have been investigated with the aim of minimizing the negative effects of excess superoxide and oxidative stress in blood vessels 21,22. Chronic aerobic training improves vasorelaxation, reduce systemic reactive oxygen species (ROS) levels, and decrease blood pressure (BP) in humans and SHR 3,. However, the mechanism by which aerobic training improves vasodilation is not yet fully understood. The vascular response to aerobic training appears to be related to an increase in NO bioavailability, which represents a balance between its production by eNOS and removal by superoxide. Therefore, the purpose of this study was to investigate the effects of aerobic training on superoxide production, NO bioavailability, and aortic endothelium-dependent vasodilation in SHR.

## MATERIALS AND METHODS

### Animals

Twelve-week-old male SHR were randomly assigned to the sedentary (SHRsd; n=10) and swimming-trained (SHRtr; n=10) groups. Age and sex-matched normotensive Wistar Kyoto rats assigned to the sedentary (WKYsd; n=10) or swimming-trained (WKYtr; n=10) were used as controls. All protocols and surgical procedures used were in accordance with the National Institutes of Health (NIH) Guide for the Care and Use of Laboratory Animals and were approved by the local ethics committee of the University of São Paulo (No 2010/15).

### Aerobic Training Protocol

Aerobic training consisted of 60-min swimming sessions, 5 days a week, for 10 weeks, with a 4% caudal body weight (BW) workload. The sessions were conducted between 11:30 A.M. and 1:30 P.M. The exercise duration and workload were gradually increased until the rats could swim for 60 min while wearing caudal dumbbells that weighed 4% of their BW. Thereafter, the swimming duration and use of dumbbell were maintained at constant levels. All animals were weighed once a week, and the workload was adjusted to BW variations. Sedentary groups were placed in the swimming apparatus for 10 min twice a week without the added workload to mimic the water stress associated with the experimental protocol. The O_2_ uptake of rats swimming individually is estimated to be approximately 50-65% of the maximum oxygen uptake (VO_2_max). This protocol was used in our previous study and is defined as a long-term moderate-intensity protocol that effectively promotes cardiovascular adaptations and increases muscle oxidative capacity 25,26.

### Graded Treadmill Exercise Test

Exercise tolerance, as measured by the total distance run, was evaluated in rats using a progressive exercise protocol on a treadmill. After being trained to run on the treadmill for one week (10 min/day), the rats were placed on the treadmill and allowed to acclimate to the apparatus for a 30-min period before beginning the progressive test. The exercise test began at 6 m/min with no grade and was increased by 3 m/min every 3 min thereafter until exhaustion was observed. WKY and SHR performed the graded treadmill exercise test before and after the experimental period 25.

### Oxygen Uptake Measurements

VO_2_ was measured using an expired gas analysis during the graded treadmill exercise test described above. The gas analysis was performed using an oxygen and carbon dioxide analyzer (Sable Systems SS3, FC-10a O_2_/CO_2_ analyzer, Las Vegas, Nevada, USA). VO_2_ was calculated based on the measured flow through the metabolic chamber, the expired fraction of effluent oxygen and the fraction of oxygen in the room air 27.

### Cardiovascular Measurements

Resting BP and heart rates (HRs) were measured in conscious rats using a computerized tail-cuff system (BP2000; Visitech System, Apex, North Carolina, USA) between 8 A.M. and 11 A.M. One week before the start of the experimental period, rats were acclimated to the apparatus during daily sessions for 4 days. We performed 5 measurements for inflation and deflation cycles, and the BP was considered the average of these values. BP and HRs were determined before and after the aerobic training protocol. The BP values were also obtained 48 h after the last exercise session.

### Preparation of Vessel Segments

Forty-eight hours after the last exercise session, the rats were euthanized by asphyxia in a carbon dioxide (100%) chamber, and the thoracic aorta was excised, cleaned of connective and/or adipose tissue, and cut into 4-mm-long rings. Two rings were promptly used to evaluate vasomotor responses *in vitro*, and the rest of the aorta was used for other measurements (eNOS, Nox1, Nox4, superoxide and nitrite levels). Each vasomotor protocol was performed simultaneously on the aortas of trained and sedentary rats.

The aortic rings utilized to determine vascular responsiveness were carefully submerged in organ bath chambers containing an oxygenated (95% O_2_ and 5% CO_2_) Krebs solution composed of (mM): NaCl 115, KCl 4.7, MgSO_4_ 1.2, KH_2_PO_4_ 1.5, NaHCO_3_ 25, CaCl_2_ 2.5, and glucose 11.1 (37°C, pH 7.4). The rings were mounted on a force transducer (BIOPAC Sytems, |Inc., Camino Goleta, California, USA) using an initial passive tension of 2.0 g, as previously determined by the maximal vasorelaxation response, and were equilibrated for a 60-min period.

### Vascular Reactivity Studies

One of the two rings from each animal was preincubated with 10^-4^ M L-NAME, an analog that inhibits NO synthase, for 30 min to investigate the effect of aerobic training on the endothelium-dependent vasorelaxation response to cumulative doses of the muscarinic receptor agonist acetylcholine (ACh: 10^–10^ to 10^–4^ M). After a 25-min washout period with warmed Krebs-bicarbonate buffer (pH 7.4, 37°C), the endothelium-independent vasorelaxation response to cumulative doses of sodium nitroprusside, an exogenous NO donor (SNP: 10^–11^ to 10^–4^ M), was assessed. All rings were precontracted with a submaximal 10^-7^ M dose of noradrenaline 28.

The data were analyzed by measuring maximum effect values (Emax values), and the concentrations that evoked 50% of the maximal effect (EC_50_ values) were estimated using an iterative nonlinear regression analysis of each animal’s concentration-response curve with GraphPad Prism Software (San Diego, California, USA).

### Molecular Analysis

The levels of the eNOS, Nox1 and Nox4 proteins in the thoracic aorta were analyzed by western blotting. The frozen aortas (20 mg) were homogenized in liquid N_2_, and the supernatants were separated by centrifugation (5,000 rpm for 5 min at 4°C) in radioimmunoprecipitation (RIPA) buffer containing 20 mM Tris-HCl, 137 mM NaCl, 1% Nonidet P-40 (NP-40), and 10% glycerol. The samples were loaded onto polyacrylamide gels (6-15%) and separated by SDS-PAGE. The percentage of the gels used depended on the molecular weight of the protein being analyzed. After electrophoresis, the proteins were electrotransferred to nitrocellulose membranes (Bio-Rad Biosciences, Berkeley, New Jersey, USA). The equal loading of samples (30 µg) and the transfer efficiency were monitored by staining the membrane with 0.5% Ponceau S. The membrane was then incubated in a blocking buffer (5% non-fat dry milk, 10 mM Tris-HCl (pH 7.6), 150 mM NaCl, and 0.1% Tween 20) for 2 h at room temperature and then incubated with a rabbit anti-Nox4 polyclonal antibody, goat anti-Nox1 polyclonal antibody, or rabbit anti-eNOS polyclonal antibody (Santa Cruz Biotechnology, Santa Cruz, California, USA) overnight at 4°C. The bound primary antibody was detected with peroxidase-conjugated secondary antibodies, and enhanced chemiluminescence reagents (Amersham Biosciences, Piscataway, New Jersey, USA) were used to visualize the autoradiogram, which was later exposed to photographic film. The film was developed, and the bands were analyzed using Scion Image software (Scion Corporation, based on NIH image). Expression of the glyceraldehyde-3-phosphate dehydrogenase (GAPDH) protein in the aorta were used to normalize the results, and the data are expressed as a percentage of the control.

### Detection of Superoxide Levels using Dihydroethidium Fluorescence Staining

Currently, the most popular probe used to measure superoxide levels with fluorescence techniques is dihydroethidium (DHE) staining. For this study, vessels were harvested from experimental animals, and 30-µm frozen sections were obtained using a cryostat. The sections were then cleaned with distilled water, incubated with DHE (10 mM for 30 min at 37°C) in a light-protected humidified chamber, and then viewed under a Zeiss fluorescence laser scanning confocal microscope (LSM 510 Meta, Carl Zeiss, Jena, Thuringia, Germany) equipped with a krypton/argon laser and a 40× objective; the same imaging settings were used in each session. Fluorescence was detected with 543 nm (DHE) and 633 nm (contrast) long-pass filters. DHE is cell permeable and reacts with superoxide to form ethidium, which in turn, intercalates with the DNA, resulting in nuclear fluorescence at an excitation wavelength of 520 nm and an emission wavelength of 610 nm. For quantification, three rings per animal were analyzed using Leica Qwin Software (Leica Microsystems, Wetzlar, Gemany), and the mean fluorescence densities in the target region were calculated 29.

### Nitrite Concentration

Vascular nitrite concentrations were measured to evaluate NO bioavailability. Vascular homogenates were prepared using liquid N_2_, centrifuged at 5,000 rpm for 5 min, and 10-μL aliquots of the supernatant were injected into a Sievers chemiluminescence analyzer (model 280; Sievers Instruments, Inc., Boulder, Colorado, USA) using VCl3 and HCl (at 95°C) as reductants as previously described 30,31. The results were normalized to the protein concentration.

### Statistical Analysis

The results are presented as means ± standard errors of the means (SEM). The statistical analysis was performed using two-way ANOVA (strain × condition). *p*-values<0.05 were considered statistically significant. *Duncan’s post hoc* test (Statistica software; StatSoft, Tulsa, OK) was used for individual comparisons between means when a significant change was observed using ANOVA. Repeated measures ANOVA was used to compare measurements obtained before and after the aerobic training period.

## RESULTS

### Hemodynamic Measurements and Aerobic Training Markers

**Figure 1:**
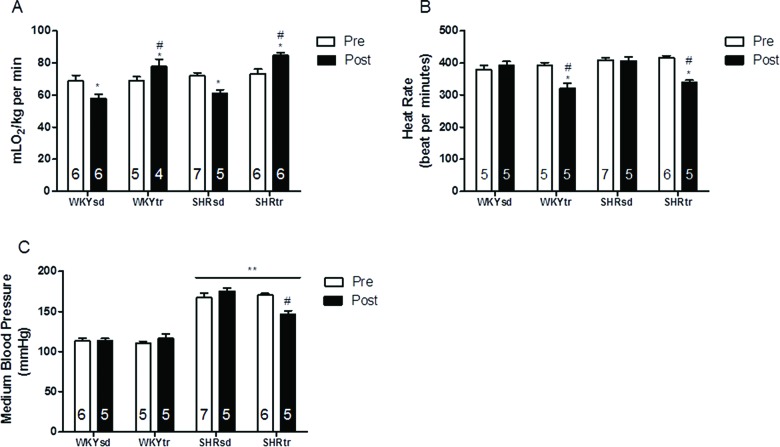
Hemodynamics and effort test variables pre- and post-aerobic training or the sedentary period. Peak oxygen uptake (A); HR (B); and MBP (C). *p<0.05; post-training compared with pre-training; #*p*<0.05; trained group after training compared with the sedentary group after training; and **p<0.01, SHR *vs*. WKY. The data are presented as the mean ± SEM.

Before starting the aerobic training protocol, all groups presented similar functional capacities (VO_2_ peak) (Figure 1A) and baseline HRs (Figure 1B). Moreover, the SHR presented a higher baseline medium BP (MBP) than the WKY rats (Figure 1C).

Aerobic training significantly improved the VO_2_ peak in the SHRtr and WKYtr groups compared with the sedentary groups (Figure 1A). Additionally, aerobic training significantly decreased the HRs and MBP of the SHRtr group compared with the SHRsd group (Figure 1B and C, respectively). The antihypertensive and bradycardic effects of swimming training have been reported previously 25.

### Endothelial Function

**Figure 2:**
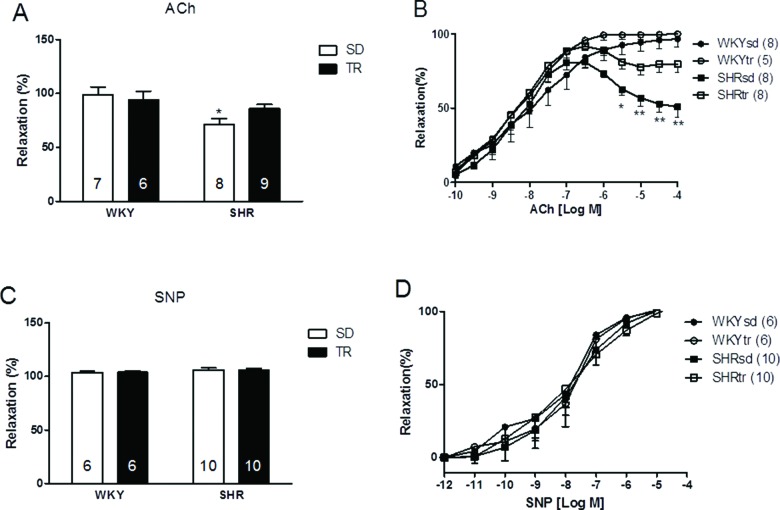
Relative maximal endothelium-dependent vasorelaxation induced by ACh (A) and relative maximal endothelium-independent vasorelaxation following the SNP treatment (C). Curves showing the maximal endothelium-dependent vasorelaxation (ACh: 10^–10^ to 10^–4^ M) and maximal endothelium-independent vasorelaxation (SNP: 10^–12^ to 10^–4^ M) (B and D, respectively) of the aortas of control WKY (circles) and SHR (square). *p<0.01 and **p<0.001 SHRsd group compared with the WKYsd, WKYtr and SHRtr groups. The data are presented as the mean ± SEM.

The relative maximal endothelium-dependent vasorelaxation (Emax) induced by acetylcholine was lower in the SHRsd group (71.6%) than in the WKYsd group (98.84%) (*p*<0.01; Figure 2A). Moreover, the endothelium-dependent vasorelaxation induced by 10^-4^ M ACh was lower in the SHRsd group than in the WKYsd group (*p*<0.001; Figure 2B). High doses of ACh induced significant vasoconstriction in the SHRsd group. Notably, aerobic training improved both the Emax and the vasodilator response to a higher dose of ACh (10^-4^ M) in the SHRtr group, which was greater than the values observed in the SHRsd group. No differences were observed between the WKYsd and WKYtr groups or between the WKY and SHRtr groups (Figure 2A and B).

Differences were not observed in the concentration that evoked 50% of the maximal effect (EC_50_ values) (Figure 2B) or the relative maximal endothelium-independent vasorelaxation induced by SNP (10^-11^ to 10^-4^ M; exogenous NO donor) between groups, thus indicating that the smooth muscle was intact in the vessels (Figures 2C and D).

**Figure 3:**
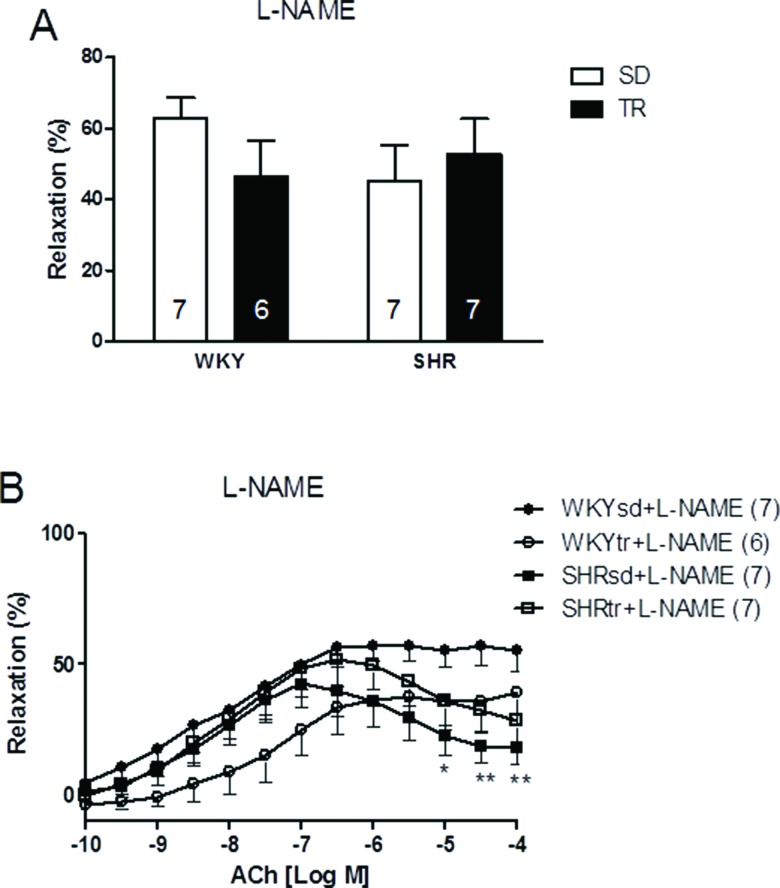
Relative maximal endothelium-dependent vasorelaxation induced by ACh (A) and curves of the maximal endothelium-dependent vasorelaxation (ACh: 10^–10^ to 10^–4^ M) of the aortas of control WKY (circles) and SHR (square) (B) after a 30-min preincubation with an inhibitor of NO synthesis (L-NAME; 10^-4^ M). *p<0.05 and **p<0.01 SHRsd compared with WKY. The data are presented as the mean ± SEM.

We inhibited eNOS-dependent relaxation to investigate whether NO production was affected by hypertension or exercise. After a 30-min treatment with a 10^-4^ M dose of the inhibitor L-NAME, the relative maximal ACh-induced vasorelaxation was similar in all groups (Figure 3A). However, after preincubation, the relative vasorelaxation induced by 10^-4^ M ACh was still significantly lower in the SHRsd group than in the WKY group (*p*<0.05; Figure 3B) and was similar to the SHRtr group. Thus, the restoration of relative maximal relaxation by exercise training in the SHR group is associated with an increase in NO bioavailability.

### Biomarkers of NO Bioavailability in the Thoracic Aorta of SHR

**Figure 4:**
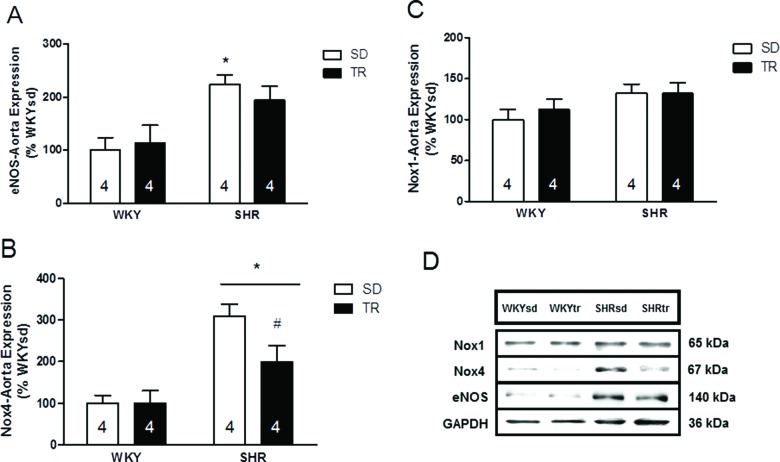
Densitometry analysis of the levels of the eNOS, *p=0.002 SHRsd group compared with WKY (A); Nox4, *p=0.001 SHR compared with WKY and #*p*=0.02 SHRtr group compared with the SHRsd group (B); and Nox1 proteins (C). Representative blots (D) of samples of the aortas from the WKYsd, WKYtr, SHRsd and SHRtr groups. The data are presented as the mean ± SEM.

The levels of enzymes controlling NO production and removal were investigated to obtain insights into the mechanisms controlling NO bioavailability. The protein levels of the endothelium-specific enzyme eNOS were analyzed by western blotting and were elevated in the SHRsd group compared to the WKY group (*p*=0.002; Figure 4A). Furthermore, the eNOS levels in the SHRtr group were similar to the levels in the WKYsd, WKYtr and SHRsd groups. Importantly, the levels of the catalytic pro-oxidant Nox4 were elevated in SHR compared to WKY (*p*=0.001; Figure 4B). However, Nox4 protein levels were lower in the SHRtr group than in the SHRsd group (*p*=0.02; Figure 4B). Significant differences in the levels of the Nox1 protein were not observed among the groups (Figure 4C). Representative blots are shown in Figure 4D.

**Figure 5:**
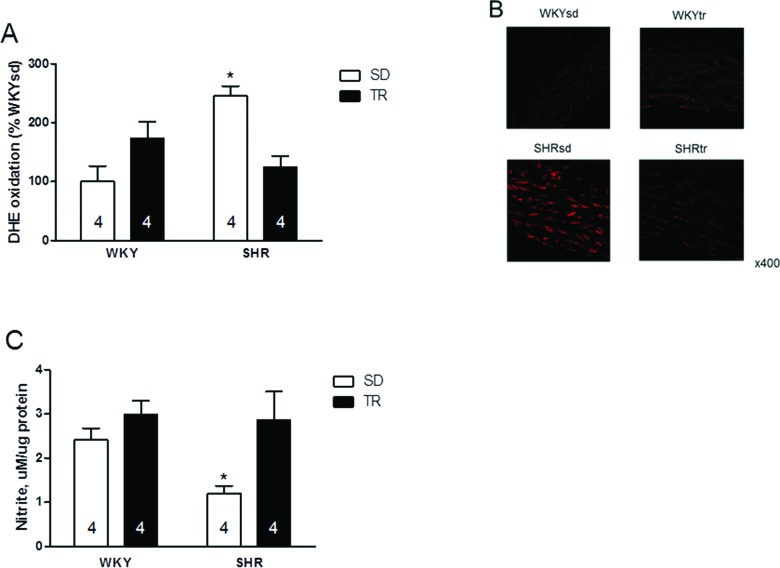
Quantitative analysis of the superoxide content, *p=0.01 SHRsd group compared with the SHRtr, WKYtr and WKYsd groups (A); representative fluorescence micrographs of confocal microscopy images (B) and vascular nitrite levels *p=0.002 SHRsd compared with the SHRtr, WKYsd and WKYtr groups (C) in samples of the aortas from the WKYsd, WKYtr, SHRsd and SHRtr groups. The data are presented as the mean ± SEM.

Furthermore, higher superoxide levels were observed in the SHRsd group than in the WKYsd, WKYtr and SHRtr groups (*p*=0.01; Figure 5A). Figure 5B shows representative photomicrographs of vascular cells stained with DHE. Interestingly, despite the increased expression of the eNOS protein, the nitrite levels were lower in the SHRsd group than in the WKYsd, WKYtr and SHRtr groups (*p*=0.02; Figure 5C).

Accordingly, aerobic training increased the nitrite content and decreased the superoxide content in the SHRtr group (*p*=0.007 and *p*=0.001 compared with the SHRsd group, respectively).

## DISCUSSION

This study aimed to examine the effects of aerobic training on endothelium-dependent vasorelaxation induced by acetylcholine and the expression of enzymes controlling NO bioavailability in the aorta of hypertensive rats.

We confirmed findings from previous reports showing that compared with the WKYsd group, the SHRsd group exhibits higher BP and impaired maximal vasorelaxation 24,25. Moreover, the relative endothelium-dependent vasorelaxation induced by 10^-4^ M ACh was lower in the SHRsd group than in WKY and showed paradoxical vasoconstriction in SHR. These results clearly reveal endothelial dysfunction in the aorta of SHR 24,32. Acetylcholine activates smooth muscle muscarinic receptors and evokes endothelium-dependent contractions in the aortas of SHR, but not WKY 33.

When aorta samples were incubated with L-NAME, differences in maximal vasorelaxation were not observed, but the relative endothelium-dependent vasorelaxation induced by ACh (10^-4^ M) remained different between the SHRsd and WKYsd groups. Therefore, the impaired vasodilation may be partially associated with NO production by eNOS. The maximal vasorelaxation induced by ACh after incubation with L-NAME was approximately 50%, consistent with the broad inhibitory effect of L-NAME on eNOS 28,34,35. Although the mechanisms underlying the variability in L-NAME effects are unclear and not addressed here, we cannot exclude the possibility that the effect was caused by loss-of-tension after noradrenaline-induced precontraction.

In addition, the increase in the production of endothelium-derived contracting factors such as prostanoids antagonizes relaxing actions and contributes to endothelial dysfunction in subjects with hypertension 36 and should be assessed in future studies.

Interestingly, the aortic levels of the eNOS protein were elevated in the SHRsd group compared to the WKYsd group, consistent with the observations of another study 24. Based on these data, the levels of the eNOS protein may be upregulated as a potential compensatory mechanism because NO bioavailability was lower in the SHRsd group than in the WKYsd group, as indicated by the lower nitrite levels in the SHRsd group. On the other hand, we cannot ignore the observation that uncoupled eNOS is also an important source of superoxide in SHR 32.

According to several classical lines of evidence, elevated vascular oxidative stress in SHR leads to impaired endothelium-dependent vasomotor function 6,7. We confirmed this hypothesis by showing that the levels of the catalytic pro-oxidant Nox4 and superoxide were elevated in the SHRsd group compared to the WKYsd group. Interestingly, a difference in Nox1 protein expression was not observed between groups, and thus, Nox4 is expressed at higher levels in the rat aorta than is Nox1 11.

Nox4 is an important signaling molecule in physiological processes since it appears to generate hydrogen peroxide, but its primary product is superoxide 15. The literature has reported beneficial roles of Nox4 in experimentally induced diabetes and atherosclerosis 37,38. However, in the pathophysiology of hypertension, such as essential hypertension, Nox4 expression seems to be upregulated by the renin-angiotensin system, which may have harmful effects. Finally, the physiological role of Nox4 is unclear, as it has been reported to have various roles in hypertension, atherosclerosis and diabetes.

Based on previous studies conducted by our group, DHE (fluorescence detection) is sensitive for evaluating the superoxide levels 28,40. Thus, the analyses of Nox4 and superoxide levels suggest the development of an increasingly pro-oxidant status in SHR that increases NO degradation.

Aerobic training has been cited as a non-pharmacological tool for the prevention and treatment of many CVDs and has been shown to modulate a variety of CVD risk factors 23,41. Based on the data presented in this study, aerobic training effectively improved the aerobic capacity and lowered the BP of SHR (Figure 1). Our results may have an important clinical impact since the present work shows different results than other studies assessing the effects of treadmill aerobic training on vascular function that did not observe significant effects on BP 24,42 and HR.

The maximum vasorelaxation was higher in the SHRtr group than in the SHRsd group, and the administration of high doses of ACh to the SHRtr group did not result in paradoxical vasoconstriction. Unlike the present study, which involved 10 weeks of physical training, the results of another study that used 8 weeks of swim training did not show improved vasodilator effects on the aorta of SHR 43. Thus, exercise duration is an extremely important factor in determining the effectiveness of aerobic training on improving the vasodilator response in SHR.

When preincubated with L-NAME, the response of the SHRtr group was similar to the SHRsd group, but different from WKY, thus suggesting the presence of additional endothelium-derived relaxing factors that are unaffected by exercise training. These results were confirmed by another study showing that the exercise-induced improvement in vasorelaxation depends on NO 28.

In contrast, differences in the expression of the eNOS protein were not observed in the SHRsd and SHRtr groups. However, eNOS activity might have been increased because eNOS activity has been shown to increase in response to acute and chronic aerobic exercise 28,42.

Treadmill training has been shown to improve ACh-induced relaxation, reduce superoxide levels, and increase nitrite levels in SHR 24,42. Here, we show for the first time that swimming training induces similar protective effects on the vasculature. Aerobic training counteracts oxidative stress in various tissues of SHR, such as the arteries, by increasing the efficiency of the antioxidant system 43,44 and thereby increasing NO bioavailability 45. Our results provide further evidence that aerobic training decreases oxidative stress in SHR, particularly the superoxide levels, and increases NO bioavailability in the aorta. This conclusion is based on the following evidence regarding the effects of aerobic swim training:

1. decreased vascular superoxide generation by Nox4;
2. decreased vascular superoxide content; and
3. increased nitrite content.

Based on the results of our study, 10 weeks of swimming training effectively restored the endothelial function in the aorta of SHR, and this response was associated with a significant increase in NO levels and a decrease in superoxide levels.

### Study Limitations

We did not investigate the role of antioxidants in response to aerobic training in our study. The different effects of aerobic training on various antioxidant enzymes may reflect the ROS levels and the basal antioxidant capacity of the tissue 44,45,47.

Although we cannot exclude the influence of antioxidant enzymes, this experiment was beyond the scope of the present study.

## AUTHOR CONTRIBUTIONS

Jordão CP, Fernandes T and Ramires PR were responsible for conception and design of the study. Jordão CP and Fernandes T were responsible for project coordination. Jordão CP, Fernandes T, Ramires PR and Oliveira EM drafted the manuscript. Jordão CP and Fernandes T were responsible for the data collection. Jordão CP and Fernandes T participated in the proteomic analysis. Jordão CP, Tanaka LY, Bechara LR and de Sousa LG were responsible for the endothelial function analyses. Jordão CP and Tanaka LY were responsible for the biochemical analyses. Jordão CP and Fernandes T were responsible for the statistical analyses. Tanaka LY, Bechara LR, de Sousa LG, Ramires PR and Oliveira EM edited and revised the manuscript.
